# 2423. Central venous catheter tip colonization and associated bloodstream infection in severe burn patients

**DOI:** 10.1093/ofid/ofad500.2042

**Published:** 2023-11-27

**Authors:** Kibum Jeon, Seung Beom Han

**Affiliations:** Hallym University Hangang Sacred Heart Hospital, Seoul, Seoul-t'ukpyolsi, Republic of Korea; Hallym University Hangang Sacred Heart Hospital, Seoul, Seoul-t'ukpyolsi, Republic of Korea

## Abstract

**Background:**

Because severe burn patients are at higher risk for catheter-related infections, routine changes of central venous catheters (CVCs) are commonly performed for them. However, there are limited information on pathogen colonization of the CVC tip and associated bloodstream infections (BSI) in burn patients.

**Methods:**

In this study, medical records of severe burn patients, in whom routine CVC changes were performed every 7 days, were retrospectively reviewed. The results of CVC tip cultures and concurrently performed blood cultures were investigated.

**Results:**

A total of 686 CVCs in 214 severe burn patients admitted to the burn intensive care unit were analyzed. A median of 2 CVCs (range: 1–15 CVCs) were inserted in each patient, and each CVC was maintained for a median of 8 days (range: 2–24 days). The median duration of catheterization was 17 days (range: 4–117 days). Of the 686 CVCs, 138 (20.1%) CVC tips were colonized by 150 pathogens (two pathogens in 12 CVCs), and 82 (59.4%) of them accompanied BSIs caused by the same pathogen. Non-flame burn (*p* < 0.001), higher total body surface area burn (*p* < 0.001), renal replacement therapy (*p* < 0.001), and femoral catheter (*p* < 0.001) were significantly associated with the development of catheter-related BSI in a multivariate analysis. Most cases of tip colonization (81.3%) and BSI (82.9%) were caused by Gram-negative bacteria: *Acinetobacter baumannii* and *Klebsiella pneumoniae* were most frequently identified.

**Conclusion:**

Catheter-related BSI was accompanied in 12.0% of the changed CVCs: nine of ten CVCs might not require routine changing. Therefore, selective CVC changing at higher risk for catheter-related BSI seems to be appropriate rather than routine CVC changing in severe burn patients. Also, source control for burn wounds should be emphasized because most common pathogens of burn wound infection caused CVC tip colonization and BSI.

**Disclosures:**

**All Authors**: No reported disclosures

